# Progesterone decline threshold in predicting early pregnancy loss: a retrospective study

**DOI:** 10.3389/fendo.2025.1611257

**Published:** 2025-09-09

**Authors:** Yanling Wei, Xiaoyu Xin, Fangxiang Mu, Fang Wang

**Affiliations:** Department of Reproductive Medicine, Lanzhou University Second Hospital, Lanzhou, China

**Keywords:** pregnancy, progesterone, early pregnancy loss, progesterone decline threshold, risk assessment

## Abstract

**Objective:**

The single measurement of serum progesterone is considered a predictor for non-viable pregnancies. However, the dynamic change in progesterone during early pregnancy loss (EPL) remains uninvestigated. This study evaluated the association between serum progesterone decline thresholds (PDT) and EPL.

**Methods:**

This retrospective study included 664 pregnant women who visited a single medical center from January 2023 to December 2024. Based on pregnancy outcomes within the first trimester, participants were classified into the ongoing pregnancy group (n=388) and the EPL group (n=286). PDT was defined as a decline of ≥ 1/5 standard deviation (SD), 1/3 SD, 1/2 SD, 7/10 SD, or 1 SD compared with the last measurement of serum progesterone levels. SD was calculated based on the baseline serum progesterone levels. Multivariate logistic regression was applied to explore the association between PDT and EPL. Receiver operating characteristic (ROC) curve analysis was conducted to assess the diagnostic value of PDT. Subgroup analyses were performed to evaluate the robustness of the results.

**Results:**

Compared with the ongoing pregnancy group, the EPL group had significantly lower baseline serum progesterone levels (P < 0.05). PDT ≥ 1/5 SD, 1/3 SD, and 1/2 SD were all significantly associated with EPL (OR [95%CI]=2.74 [1.76, 4.27], P < 0.001; OR [95%CI]=1.74 [1.18, 2.56], P=0.005; and OR [95%CI]=1.63 [1.07, 2.49], P=0.024, respectively). The corresponding AUC values were 0.502, 0.512, and 0.503. Additionally, a linear positive correlation was observed between the number of occurrences of PDT ≥ 1/3 SD and EPL. For each additional occurrence of PDT ≥ 1/3 SD, the risk of EPL increased by 36% (OR [95%CI]=1.36 [1.09, 1.70], P=0.006). Subgroup analyses supported the robustness of these results.

**Conclusion:**

PDT ≥ 1/5 SD, 1/3 SD, and 1/2 SD are significantly associated with an increased risk of EPL. This suggests that these thresholds hold potential predictive value in EPL diagnosis and may help identify pregnant women at higher risk for early intervention.

## 1 Introduction

Pregnancy loss affects 15%–25% of clinically recognized pregnancies (1). Approximately 80% of pregnancy losses occur during the first trimester (up to 12 weeks and 6/7 days), termed early pregnancy loss (EPL) (2). Common symptoms of EPL include vaginal bleeding and uterine cramping (3); however, these symptoms are also observed in normal and ectopic pregnancies, which makes the diagnosis and management of EPL challenging.

Transvaginal ultrasonographic (TVS) diagnosis is the primary method for confirming EPL by detecting fetal cardiac activity. However, due to incomplete embryonic development in early pregnancy, a single TVS examination may not provide a definitive diagnosis, often requiring follow-up scans within 7–14 days (4–6). Consequently, researchers have focused on identifying highly sensitive and specific biomarkers for early EPL diagnosis (7–9).

Progesterone is secreted by the corpus luteum, which ensures normal embryonic development by establishing maternal-fetal immune tolerance, inhibiting uterine contractions, and improving uteroplacental circulation (10). Its levels remain relatively stable before 9 weeks of gestation and gradually increase after 10–12 weeks as the placenta takes over secretion (11). Studies have consistently confirmed that serum progesterone levels are significantly lower in women experiencing pregnancy loss compared to those with ongoing pregnancies (12, 13), and baseline progesterone levels in early pregnancy have been shown to aid in discriminating between viable and non-viable pregnancies (14–16). However, progesterone levels fluctuate significantly within individuals due to pulsatile secretion patterns, hormone distribution, and dietary influences (17–19), particularly when gestational age is not consistently recorded in studies. Furthermore, studies have suggested that the progesterone level partially overlaps between normal and abnormal pregnancies (20, 21), which complicates their clinical application.

Therefore, we hypothesize that the dynamic monitoring of serum progesterone decline might address the limitations of single measurements because it captures changes in progesterone levels between measurements. In this study, we aimed to evaluate the association between progesterone decline threshold (PDT) and EPL, which may provide predictive value for EPL diagnosis.

## 2 Methods

### 2.1 Participants

This retrospective analysis was conducted on 1,865 pregnant women who visited the Department of Reproductive Medicine, Lanzhou University Second Hospital between January 2023 and December 2024. The study was approved by the Ethics Committee of Lanzhou University Second Hospital (Approval No. 2019A-231), and all participants provided written informed consent.

The inclusion criteria were as follows (1): Age between 18 and 45 years (2); Natural conception (3); Availability of early pregnancy outcome (4); At least two progesterone measurements completed between 3 and 12 weeks of pregnancy.

Participants were excluded if they had (1): Parental or embryonic chromosomal abnormalities (2); Congenital uterine anomalies (e.g., septate uterus, unicornuate uterus, bicornuate uterus, or uterus didelphys) without surgical correction during the current pregnancy (3); Multiple pregnancies (4); Infertility (5); Ectopic pregnancy (6); Missing progesterone data or fewer than two measurements. The participants’ demographics were also recorded, including maternal age, body mass index (BMI), age at menarche, and menstrual regularity.

### 2.2 Progesterone measurement

Progesterone (ng/mL) was the exposure variable in this study. Peripheral venous blood was collected from all patients during their visits, and serum was separated after centrifugation. Progesterone levels were measured using an automated chemiluminescence immunoassay analyzer (Immulite 1000, Siemens Healthineers). The timing of subsequent measurements was determined based on pregnancy status.

### 2.3 Definition of PDT

In this study, any progesterone level lower than the last measurement was considered a decline. To explore the association between PDT and EPL, PDT was defined as a decline of ≥ 1/5 standard deviation (SD), 1/3 SD, 1/2 SD, 7/10 SD, and 1 SD compared to the last measurement of serum progesterone levels. SD was calculated from the baseline serum progesterone levels of the eligible participants.

### 2.4 Study outcomes

The study outcomes were pregnancy loss (including biochemical pregnancy) and ongoing pregnancy within 12 weeks of gestation. Embryonic viability was assessed using TVS. Ongoing pregnancy was defined as the presence of embryonic cardiac activity, while EPL was defined as the absence of cardiac activity, confirmed by repeated TVS after 7–14 days.

### 2.5 Statistical analysis

Categorical variables were presented as numbers and percentages (%), and group comparisons were performed using the Chi-square test. Continuous variables, if normally distributed, were described as mean ± SD; otherwise, they were presented as median (interquartile range). Group differences for continuous variables were compared using the Student’s t-test or the Kruskal-Wallis H test. Missing data were handled using multiple imputation.

The association between PDT and EPL was investigated using multivariate logistic regression analysis. To control for confounding factors, three adjusted models were constructed: Model 1 adjusted for age; Model 2 adjusted for age and baseline serum progesterone; Model 3 adjusted for age, baseline serum progesterone, and number of progesterone measurements. Receiver operator characteristic (ROC) curve analysis was conducted to assess the diagnostic value of PDT, and the area under the ROC curve (AUC) was calculated using the DeLong test. Restricted cubic spline analysis was utilized to explore potential non-linear relationships between the number of PDT occurrences and EPL. Furthermore, subgroup analyses were performed by dividing participants into two groups based on whether their progesterone levels fell below baseline, to assess the robustness of the association between PDT and EPL.

All statistical analyses were conducted using R version 4.3.1 (http://www.R-project.org, The R Foundation) and EmpowerStats version 4.2 (https://www.empowerstats.net/en/; X&Y solutions, Inc.). All statistical tests were two-tailed, with P < 0.05 considered statistically significant.

## 3 Results

### 3.1 Baseline characteristics of the participants

**Figure 1:**
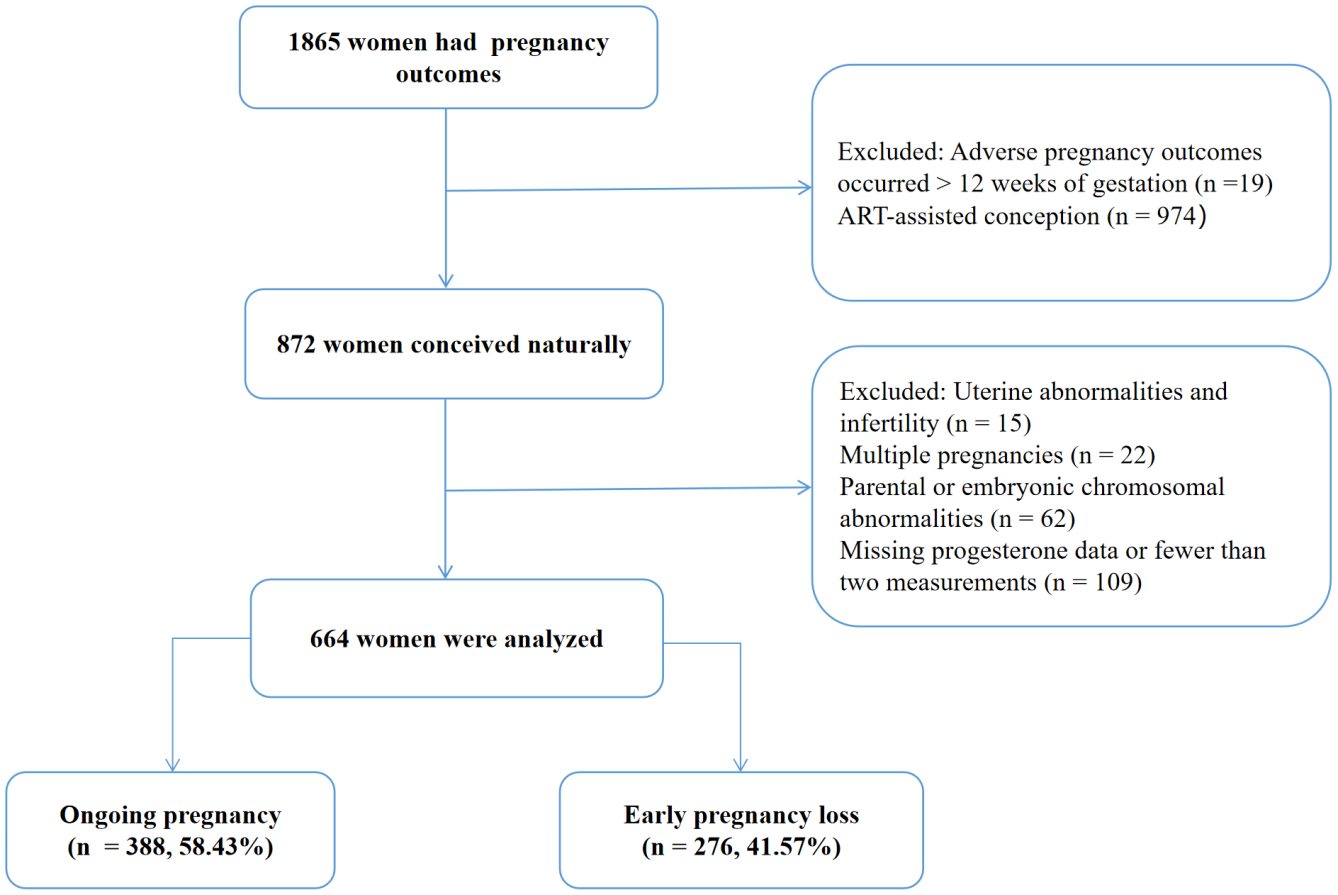
Participant selection flow diagram.

This study included 664 women who met the inclusion criteria (**Figure 1**), with 276 cases of EPL and 388 cases of ongoing pregnancy. **Table 1** summarizes the characteristics of the participants. The EPL group had significantly higher age (31.56 ± 3.98 vs. 30.62 ± 3.70 years, P=0.004) and body mass index (22.04 ± 2.95 vs. 21.52 ± 2.92 kg/m², P=0.018), as well as significantly lower number of progesterone measurements (5.84 ± 3.42 vs. 8.78 ± 3.78, P < 0.001) and baseline serum progesterone levels (30.73 ± 24.74 vs. 35.62 ± 25.47 ng/mL, P < 0.001) compared to the ongoing pregnancy group (all P < 0.05). Other characteristics were comparable between the two groups.

**Table 1:** Baseline characteristics of participants.

Characteristics	Total (n=664)	Ongoing pregnancy (n=388)	EPL (n=276)	P value
Age, years, mean ± SD	31.01 ± 3.85	30.62 ± 3.70	31.56 ± 3.98	0.004
BMI, kg/m^2^, mean ± SD	21.73 ± 2.94	21.52 ± 2.92	22.04 ± 2.95	0.018
Age at menarche, years, mean ± SD	13.00 ± 1.33	12.98 ± 1.22	13.02 ± 1.47	0.747
Baseline serum progesterone, ng/mL, mean ± SD	33.59 ± 25.27	35.62 ± 25.47	30.73 ± 24.74	<0.001
Number of progesterone measurements, mean ± SD	7.56 ± 3.91	8.78 ± 3.78	5.84 ± 3.42	<0.001
Regularity of menstruation, n (%)				0.690
No	147 (22.14%)	88 (22.68%)	59 (21.38%)	
Yes	517 (77.86%)	300 (77.32%)	217 (78.62%)	
Serum progesterone below baseline, n (%)				0.615
No	98 (14.76%)	55 (14.18%)	43 (15.58%)	
Yes	566 (85.24%)	333 (85.82%)	233 (84.42%)	
Serum progesterone declined, n (%)				0.056
No	31 (4.67%)	13 (3.35%)	18 (6.52%)	
Yes	633 (95.33%)	375 (96.65%)	258 (93.48%)	
PDT ≥ 1/5 SD, n (%)				0.905
No	170 (25.60%)	100 (25.77%)	70 (25.36%)	
Yes	494 (74.40%)	288 (74.23%)	206 (74.64%)	
PDT ≥ 1/3 SD, n (%)				0.556
No	311 (46.84%)	178 (45.88%)	133 (48.19%)	
Yes	353 (53.16%)	210 (54.12%)	143 (51.81%)	
PDT ≥ 1/2 SD, n (%)				0.849
No	455 (68.52%)	267 (68.81%)	188 (68.12%)	
Yes	209 (31.48%)	121 (31.19%)	88 (31.88%)	
PDT ≥ 7/10 SD, n (%)				0.870
No	520 (78.31%)	303 (78.09%)	217 (78.62%)	
Yes	144 (21.69%)	85 (21.91%)	59 (21.38%)	
PDT ≥ 1 SD, n (%)				0.095
No	551 (82.98%)	314 (80.93%)	237 (85.87%)	
Yes	113 (17.02%)	74 (19.07%)	39 (14.13%)	

EPL, early pregnancy loss; SD, standard deviation; BMI, body mass index; PDT, progesterone decline threshold.

### 3.2 Association between PDT and EPL

**Figure 2:**
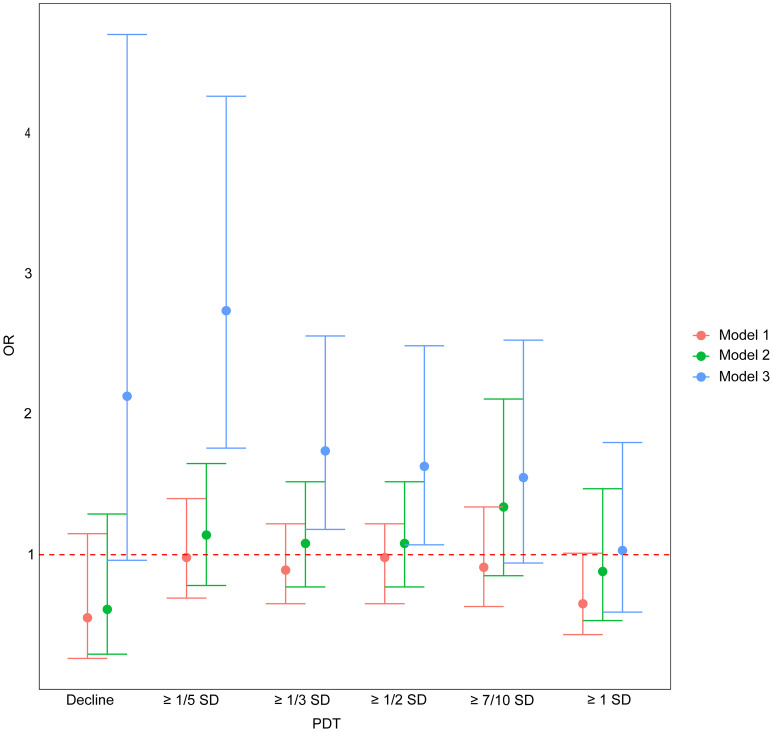
The association between PDT and EPL. OR, odds ratio; PDT, progesterone decline threshold; SD, standard deviation. Model 1: adjusted for age; Model 2: adjusted for age and baseline serum progesterone; Model 3: adjusted for age, baseline serum progesterone, and number of progesterone measurements.

In Models 1 and 2, PDT was not significantly associated with EPL (P > 0.05). In Model 3, however, PDT ≥ 1/5 SD, 1/3 SD, and 1/2 SD were positively associated with EPL, with risks of 2.74-fold (95%CI: 1.76, 4.27, P < 0.001), 1.74-fold (95%CI: 1.18, 2.56, P=0.005), and 1.63-fold (95%CI: 1.07, 2.49, P=0.024) for those experiencing a decline compared to those without a decline, respectively (**Table 2**, **Figure 2**).

**Table 2:** The association between PDT and EPL.

Exposure	Model 1 OR (95%CI)	P value	Model 2 OR (95%CI)	P value	Model 3 OR (95%CI)	P value
Serum progesterone declined						
No	Reference	—	Reference	—	Reference	—
Yes	0.55 (0.26, 1.15)	0.113	0.61 (0.29, 1.29)	0.195	2.13 (0.96, 4.71)	0.063
PDT ≥ 1/5 SD						
No	Reference	—	Reference	—	Reference	—
Yes	0.98 (0.69, 1.40)	0.919	1.14 (0.78, 1.65)	0.498	2.74 (1.76, 4.27)	<0.001
PDT ≥ 1/3 SD						
No	Reference	—	Reference	—	Reference	—
Yes	0.89 (0.65, 1.22)	0.470	1.08 (0.77, 1.52)	0.664	1.74 (1.18, 2.56)	0.005
PDT ≥ 1/2 SD						
No	Reference	—	Reference	—	Reference	—
Yes	0.98 (0.70, 1.38)	0.929	1.33 (0.90, 1.96)	0.149	1.63 (1.07, 2.49)	0.024
PDT ≥ 7/10 SD						
No	Reference	—	Reference	—	Reference	—
Yes	0.91 (0.63, 1.34)	0.642	1.34 (0.85, 2.11)	0.210	1.55 (0.94, 2.53)	0.083
PDT ≥ 1 SD						
No	Reference	—	Reference	—	Reference	—
Yes	0.65 (0.43, 1.01)	0.053	0.88 (0.53, 1.47)	0.625	1.03 (0.59, 1.80)	0.910

OR, odds ratio; CI, confidence interval; PDT, progesterone decline threshold; SD, standard deviation.

Model 1: adjusted for age.

Model 2: adjusted for age and baseline serum progesterone.

Model 3: adjusted for age, baseline serum progesterone, and number of progesterone measurements.

### 3.3 Diagnostic value of PDT

The ROC analysis showed that when PDT ≥ 1/5 SD, 1/3 SD, and 1/2 SD, the AUC values were 0.502, 0.512, and 0.503, respectively (**Table 3**). The DeLong test results were not significant (P > 0.05), indicating no significant differences in diagnostic performance for EPL across these decline magnitudes.

**Table 3:** ROC analysis of the predictive value of PDT in predicting EPL.

Exposure	Area of ROC	95%CI	Z	P value
PDT ≥ 1/5 SD	0.502	0.468, 0.536	-0.286	0.775^a^
PDT ≥ 1/3 SD	0.512	0.473, 0.550	0.234	0.815^b^
PDT ≥ 1/2 SD	0.503	0.468, 0.539	-0.074	0.941^c^

ROC, Receiver operating characteristic; PDT, progesterone decline threshold; EPL, early pregnancy loss; SD, standard deviation. AUC, Area under curve.

a PDT ≥1/5 SD vs PDT ≥1/3 SD;

b PDT ≥1/3SD vs PDT ≥ 1/2 SD;

c PDT ≥1/5 SD vs PDT ≥ 1/2 SD.

### 3.4 Association between the number of occurrences of PDT ≥ 1/3 SD and EPL

**Figure 3:**
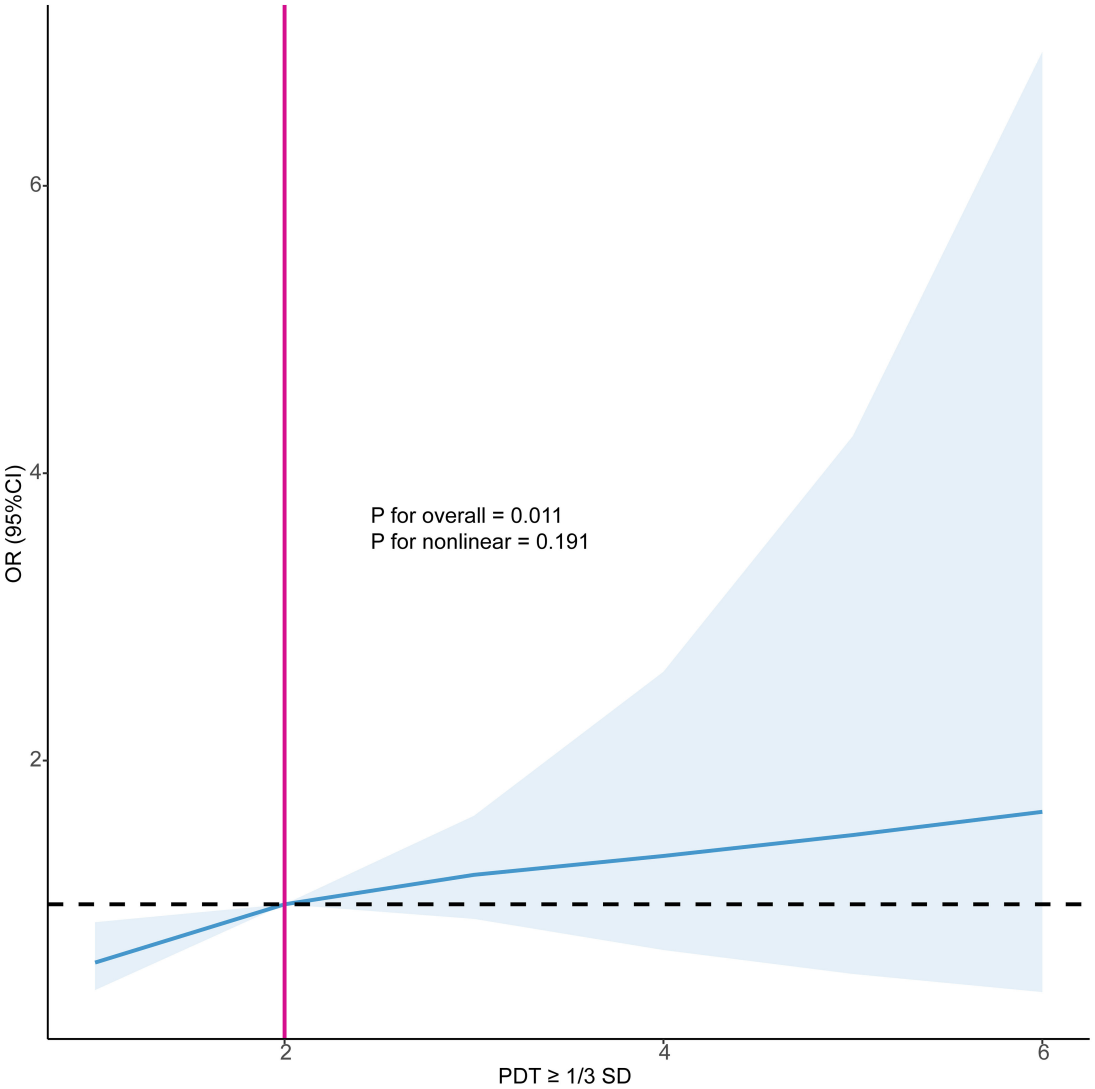
Restricted cubic splines examined the association between the number of occurrences of PDT ≥ 1/3 SD and EPL. PDT, progesterone decline threshold; SD, standard deviation; OR, odds ratio; CI, confidence interval.

The logistic regression results presented in **Table 4** indicated that, in Model 3, the number of occurrences of PDT ≥ 1/3 SD was significantly positively associated with EPL. Each additional occurrence of this decline increased the risk of EPL by 36% (OR=1.36, 95%CI: 1.09, 1.70, P=0.006). Similarly, restricted cubic spline analysis also demonstrated a linear association between the number of occurrences of PDT ≥ 1/3 SD and EPL (P < 0.05), with an increasing risk of EPL as the occurrences of declines increased (**Figure 3**).

**Table 4:** The association between the number of occurrences of PDT ≥ 1/3 SD and EPL.

Exposure	Model 1 OR (95%CI)	P value	Model 2 OR (95%CI)	P value	Model 3 OR (95%CI)	P value
Number of occurrences	0.84 (0.71,0.98)	0.034	0.92 (0.76,1.11)	0.376	1.36 (1.09,1.70)	0.006

PDT, progesterone decline threshold; EPL, early pregnancy loss; SD, standard deviation; OR, odds ratio; CI, confidence interval.

Model 1: adjusted for age.

Model 2: adjusted for age and baseline serum progesterone.

Model 3: adjusted for age, baseline serum progesterone, and number of progesterone measurements.

### 3.5 Subgroup analysis

When PDT ≥ 1/5 SD and 1/3 SD, along with the serum progesterone levels below baseline levels, they were significantly positively associated with EPL risk (OR=2.76, 95%CI: 1.66, 4.60, P < 0.001 for 1/5SD; OR=1.54, 95%CI: 1.01, 2.34, P=0.043 for 1/3 SD) (**Table 5**). However, no significant associations with EPL were observed in the other subgroups. Additionally, all P for interaction values across subgroups were not significant, suggesting that the significant associations between PDT ≥ 1/5 SD, 1/3 SD, 1/2 SD, and EPL were robust regardless of whether the serum progesterone levels after decline were below baseline.

**Table 5:** Subgroup analyses of the effect of PDT on EPL.

Subgroups	PDT	n	OR (95%CI)	P value	P for interaction
Serum progesterone below baseline	PDT ≥ 1/5 SD				0.441
No	No	54	Reference	—	
	Yes	44	2.08 (0.72,5.98)	0.174	
Yes	No	116	Reference	—	
	Yes	450	2.76 (1.66,4.60)	<0.001	
	PDT ≥ 1/3 SD				0.386
No	No	70	Reference	—	
	Yes	28	2.87 (0.96,8.64)	0.060	
Yes	No	241	Reference	—	
	Yes	325	1.54 (1.01,2.34)	0.043	
	PDT ≥ 1/2 SD				0.340
No	No	83	Reference	—	
	Yes	15	3.19 (0.89,11.49)	0.076	
Yes	No	372	Reference	—	
	Yes	194	1.47 (0.93,2.33)	0.102	

PDT, progesterone decline threshold; EPL, early pregnancy loss; SD, standard deviation; OR, odds ratio; CI, confidence interval.

## 4 Discussion

Developing useful and reliable clinical prediction models based on serum biomarkers is crucial for identifying at-risk populations to improve their pregnancy outcomes. To our knowledge, this study is the first to investigate the predictive value of varying decline thresholds of progesterone levels for EPL. The results indicate that changes in progesterone levels can effectively predict pregnancy outcomes. Specifically, when PDT ≥ 1/5 SD, 1/3 SD, and 1/2 SD, the risk of EPL increased by 2.74 times, 1.76 times, and 1.63 times, respectively. Furthermore, each additional occurrence of PDT ≥ 1/3 SD increased the risk of EPL by 36%. These findings remained robust even when serum progesterone levels, after a decline, were below baseline levels. This study supports that PDT ≥ 1/5 SD, 1/3 SD, and 1/2 SD holds potential predictive value in EPL diagnosis, which may aid clinicians in developing more targeted interventions.

Progesterone levels fluctuate and rise during pregnancy, playing a critical role in maintaining gestation. A decrease in progesterone levels in early pregnancy may reflect inadequate luteal function or abnormal placental development, leading to compromised pregnancy maintenance (22, 23). The lower the serum progesterone levels, the lower the likelihood of pregnancy viability (24). Therefore, previous studies have sought to identify a progesterone cut-off value for predicting pregnancy outcome. Hanita et al., (25) Li et al., (26) and Puget etal., (27) reported cut-off values of 32.7, 19.4, and 6.2 ng/mL ng/mL, respectively, for predicting non-viable pregnancies. However, Sakar reported that a 10.7 ng/mL cut-off value more accurately identified viable pregnancies but poorly diagnosed non-viable ones (28). Additionally, some studies noted that the diagnostic cut-off value for non-viable pregnancies might be influenced by gestational age and symptoms (e.g., bleeding or pain) (29, 30). Collectively, inter-study heterogeneity complicates the selection of a reliable cut-off value. In contrast, this study introduces dynamic monitoring of serum progesterone changes and suggests that PDT ≥ 1/5 SD, 1/3 SD, and 1/2 SD has predictive value for diagnosing EPL. This approach minimizes bias from assay variability and population differences, allows earlier prediction of EPL, and may improve clinical decision-making and management.

Dynamic monitoring of early pregnancy hormones is clinically valuable for assessing gestational outcomes. Whittaker et al. performed serial measurements of progesterone, estradiol, and human chorionic gonadotropin (hCG) from gestational days 21 to 91 in asymptomatic women who later experienced early pregnancy failure. They observed that, around day 50, hormone levels continued to rise in normal pregnancies but declined in the early-failure group, indicating that dynamic monitoring can identify high-risk, asymptomatic women earlier (7). Similarly, Li et al. showed that tracking changes in estradiol and hCG over time enabled earlier detection of bad pregnancy outcomes (31). Additionally, Mu et al. reported that the average estradiol decreased times correlated positively with EPL risk (32). Su et al. further examined the absolute rate of progesterone change (Δprogesterone) between weeks 6 and 10, finding it predictive of outcome—though not as strongly as ΔhCG or Δestradiol (33). Our findings also support the significance of dynamic monitoring for pregnancy progress. We define PDT to stratify declines between consecutive measurements and suggest its significant predictive value for EPL.

Although our findings have positive clinical implications for managing pregnant women, they should be interpreted with caution. We observed that as PDT increased, the associated risk of EPL weakened, and PDT ≥ 7/10 SD and 1 SD did not show significance. This may be due to the reduced sample size of participants with progesterone decline participants as PDT increased, leading to lower statistical power. Future studies should aim to expand sample sizes and adopt prospective designs to more accurately assess the relationship between PDT and the risk of EPL. Additionally, our model’s AUC values were relatively low (0.502, 0.503, and 0.512), indicating limited predictive ability on their own. This may reflect progesterone’s intrinsic pulsatile secretion, in which a single decline may indicate physiological fluctuations rather than a pathological state; furthermore, our study did not control the intervals between measurements, inherently introducing risks of information bias. As a result, in clinical practice, these PDT indicators should serve as supplementary or adjunctive diagnostic tools. For example, combining other available indicators, such as new ultrasonographic parameters, hCG, estradiol, and PAPP-A (31, 34), to conduct multivariate analysis could enhance the diagnostic performance of the predictive models. Moreover, this study was retrospective and relied on historical case data, excluding patients with incomplete progesterone testing records, which inherently introduced risks of selection bias and potential information bias. In addition, we did not obtain medication information for these participants, making it impossible to exclude the use of progesterone supplements, which affects the accuracy of the results. Finally, as these results lack external validation, we plan to confirm their reliability in larger, independent, multicenter cohorts.

In conclusion, our study suggests that PDT ≥ 1/5 SD, 1/3 SD, and 1/2 SD may provide useful information for identifying high-risk EPL populations, thereby assisting clinical decision-making. Currently, EPL diagnosis primarily relies on TVS examinations, which have limited utility in early pregnancy due to incomplete embryonic development. Our findings offer a potential complementary tool for this diagnostic process. By incorporating dynamic assessments of progesterone levels, particularly cases where serum progesterone decline exceeds 1/5 SD, 1/3 SD, and 1/2 SD, clinicians may identify high-risk EPL populations earlier. This could enable more frequent pregnancy monitoring or early interventions, providing scientifically based decision support for early screening and management of EPL.

## 5 Conclusion

PDT ≥ 1/5 SD, 1/3 SD, and 1/2 SD significantly increased the risk of EPL. These results may aid in EPL diagnosis and help clinicians optimize pregnancy management strategies. Future large-scale studies are needed to further validate the application value of these findings.

## Data Availability

The raw data supporting the conclusions of this article will be made available by the authors, without undue reservation.

## References

[1] Practice Committee of the American Society for Reproductive Medicine. Evaluation and treatment of recurrent pregnancy loss: a committee opinion. Fertility sterility. (2012) 98:1103–11. doi: 10.1016/j.fertnstert.2012.06.048, PMID: 22835448

[2] American College of Obstetricians and Gynecologists’ Committee on Practice Bulletins—Gynecology ACOG practice bulletin no. 200: early pregnancy loss. Obstet Gynecol. (2018) 132:e197–207. doi: 10.1097/AOG.0000000000002899, PMID: 30157093

[3] Sapra KJ Joseph KS Galea S Bates LM Louis GM Ananth CV. Signs and symptoms of early pregnancy loss. Reprod Sci (Thousand Oaks Calif). (2017) 24:502–13. doi: 10.1177/1933719116654994, PMID: 27342274 PMC5933199

[4] Preisler J Kopeika J Ismail L Vathanan V Farren J Abdallah Y. Defining safe criteria to diagnose miscarriage: prospective observational multicenter study. BMJ (Clinical Res ed). (2015) 351:h4579. doi: 10.1136/bmj.h4579, PMID: 26400869 PMC4580727

[5] Huchon C Deffieux X Beucher G Capmas P Carcopino X Costedoat-Chalumeau N. Pregnancy loss: French clinical practice guidelines. Eur J obstetrics gynecology Reprod Biol. (2016) 201:18–26. doi: 10.1016/j.ejogrb.2016.02.015, PMID: 27039249

[6] Doubilet PM Benson CB Bourne T Blaivas M Barnhart KT Benacerraf BR. Diagnostic criteria for nonviable pregnancy early in the first trimester. New Engl J Med. (2013) 369:1443–51. doi: 10.1056/NEJMra1302417, PMID: 24106937

[7] Whittaker PG Schreiber CA Sammel MD. Gestational hormone trajectories and early pregnancy failure: a reassessment. Reprod Biol endocrinology: RB&E. (2018) 16:95. doi: 10.1186/s12958-018-0415-1, PMID: 30309358 PMC6182860

[8] Huang J Lv P Lian Y Zhang M Ge X Li S. Construction of machine learning tools to predict threatened miscarriage in the first trimester based on AEA, progesterone and β-hCG in China: a multicenter, observational, case-control study. BMC Pregnancy Childbirth. (2022) 22:697. doi: 10.1186/s12884-022-05025-y, PMID: 36085038 PMC9461209

[9] Pillai RN Konje JC Tincello DG Potdar N. Role of serum biomarkers in the prediction of outcome in women with threatened miscarriage: a systematic review and diagnostic accuracy meta-analysis. Hum Reprod update. (2016) 22:228–39. doi: 10.1093/humupd/dmv054, PMID: 26663220

[10] Di Renzo GC Giardina I Clerici G Brillo E Gerli S. Progesterone in normal and pathological pregnancy. Hormone Mol Biol Clin Invest. (2016) 27:35–48. doi: 10.1515/hmbci-2016-0038, PMID: 27662646

[11] Taraborrelli S. Physiology, production and action of progesterone. Acta obstetricia gynecologica Scandinavica. (2015) 94 Suppl 161:8–16. doi: 10.1111/aogs.12771, PMID: 26358238

[12] Duan L Yan D Zeng W Yang X Wei Q. Predictive power progesterone combined with beta human chorionic gonadotropin measurements in the outcome of threatened miscarriage. Arch gynecology obstetrics. (2011) 283:431–5. doi: 10.1007/s00404-010-1367-7, PMID: 20107822

[13] Lek SM Ku CW Allen JC Jr. Malhotra R Tan NS Østbye T. Validation of serum progesterone <35nmol/L as a predictor of miscarriage among women with threatened miscarriage. BMC Pregnancy Childbirth. (2017) 17:78. doi: 10.1186/s12884-017-1261-4, PMID: 28264669 PMC5340043

[14] Yalçin I Taşkin S Pabuçcu EG Söylemez F. The value of placental protein 13, β-human chorionic gonadotropin and progesterone in the prediction of miscarriages in threatened miscarriage patients. J obstetrics gynecology. (2015) 35:283–6. doi: 10.3109/01443615.2014.948822, PMID: 25153203

[15] Ucyigit A Fuller JL Poon LC Johns J Ross JA. The significance of low first trimester serum progesterone in ongoing early pregnancies presenting as pregnancies of unknown location. Eur J obstetrics gynecology Reprod Biol. (2021) 258:294–8. doi: 10.1016/j.ejogrb.2021.01.013, PMID: 33498002

[16] He S Allen JC Jr. Malhotra R Østbye T Tan TC. Association of maternal serum progesterone in early pregnancy with low birth weight and other adverse pregnancy outcomes. J maternal-fetal neonatal Med. (2016) 29:1999–2004. doi: 10.3109/14767058.2015.1072159, PMID: 26335272

[17] Filicori M Butler JP Crowley WF Jr. Neuroendocrine regulation of the corpus luteum in the human. Evidence for pulsatile progesterone secretion. J Clin Invest. (1984) 73:1638–47. doi: 10.1172/JCI111370, PMID: 6427277 PMC437074

[18] Schliep KC Mumford SL Hammoud AO Stanford JB Kissell KA Sjaarda LA. Luteal phase deficiency in regularly menstruating women: prevalence and overlap in identification based on clinical and biochemical diagnostic criteria. J Clin Endocrinol Metab. (2014) 99:E1007–14. doi: 10.1210/jc.2013-3534, PMID: 24606080 PMC4037737

[19] Nakajima ST McAuliffe T Gibson M. The 24-hour pattern of the levels of serum progesterone and immunoreactive human chorionic gonadotropin in normal early pregnancy. J Clin Endocrinol Metab. (1990) 71:345–53. doi: 10.1210/jcem-71-2-345, PMID: 2199478

[20] Radwanska E Frankenberg J Allen EI. Plasma progesterone levels in normal and abnormal early human pregnancy. Fertility sterility. (1978) 30:398–402. doi: 10.1016/S0015-0282(16)43571-5, PMID: 710610

[21] Ku CW Allen JC Jr. Lek SM Chia ML Tan NS Tan TC. Serum progesterone distribution in normal pregnancies compared to pregnancies complicated by threatened miscarriage from 5 to 13 weeks gestation: a prospective cohort study. BMC Pregnancy Childbirth. (2018) 18:360. doi: 10.1186/s12884-018-2002-z, PMID: 30185145 PMC6126027

[22] Shah D Nagarajan N. Luteal insufficiency in first trimester. Indian J Endocrinol Metab. (2013) 17:44–9. doi: 10.4103/2230-8210.107834, PMID: 23776852 PMC3659905

[23] Tuckey RC. Progesterone synthesis by the human placenta. Placenta. (2005) 26:273–81. doi: 10.1016/j.placenta.2004.06.012, PMID: 15823613

[24] Bobdiwala S Kyriacou C Christodoulou E Farren J Mitchell-Jones N Al-Memar M. Evaluating cut-off levels for progesterone, β human chorionic gonadotropin and β human chorionic gonadotropin ratio to exclude pregnancy viability in women with a pregnancy of unknown location: A prospective multicenter cohort study. Acta obstetricia gynecologica Scandinavica. (2022) 101:46–55. doi: 10.1111/aogs.14295, PMID: 34817062 PMC9564682

[25] Hanita O Hanisah AH. Potential use of single measurement of serum progesterone in detecting early pregnancy failure. Malaysian J pathology. (2012) 34:41–6. doi: 10.1002/jcla.23559, PMID: 22870597

[26] Li H Qin S Xiao F Li Y Gao Y Zhang J. Predicting first-trimester outcome of embryos with cardiac activity in women with recurrent spontaneous abortion. J Int Med Res. (2020) 48:300060520911829. doi: 10.1177/0300060520911829, PMID: 32527173 PMC7294372

[27] Puget C Joueidi Y Bauville E Laviolle B Bendavid C Lavoué V. Serial hCG and progesterone levels to predict early pregnancy outcomes in pregnancies of uncertain viability: A prospective study. Eur J obstetrics gynecology Reprod Biol. (2018) 220:100–5. doi: 10.1016/j.ejogrb.2017.11.020, PMID: 29202392

[28] Sakar MN Balsak D Demir SS Budak MŞ Tahaoglu AE Gungor SE. The ability of a single serum progesterone measurement to predict the prognosis of first trimester pregnancy. Gynecology Obstetrics Reprod Med. (2020) 26:1–5. doi: 10.21613/GORM.2019.942

[29] Deng W Sun R Du J Wu X Ma L Wang M. Prediction of miscarriage in first trimester by serum estradiol, progesterone and β-human chorionic gonadotropin within 9 weeks of gestation. BMC Pregnancy Childbirth. (2022) 22:112. doi: 10.1186/s12884-021-04158-w, PMID: 35144584 PMC8832762

[30] Verhaegen J Gallos ID van Mello NM Abdel-Aziz M Takwoingi Y Harb H. Accuracy of single progesterone test to predict early pregnancy outcome in women with pain or bleeding: meta-analysis of cohort studies. BMJ (Clinical Res ed). (2012) 345:e6077. doi: 10.1136/bmj.e6077, PMID: 23045257 PMC3460254

[31] Li Y Zhang J Zhang K Wang E Shu J. Significance of dynamically monitoring serum estrogen and β-human chorionic gonadotropin in early pregnancy assessment. J Clin Lab analysis. (2021) 35:e23559. doi: 10.1002/jcla.23559, PMID: 32892443 PMC7843287

[32] Mu F Wang C Li X Wang F. The relationship between the average decreased times of estradiol and early miscarriage: an observational study. Reprod Sci (Thousand Oaks Calif). (2025) 32:358–65. doi: 10.1186/s12884-021-04158-w, PMID: 38710977 PMC11825642

[33] Su R Wang Y Lu Y Lin B An J. Weekly changes in serum β-human chorionic gonadotropin, estradiol, and progesterone levels for pregnancy assessment in women with unexplained recurrent miscarriage. J Int Med Res. (2025) 53:3000605251327478. doi: 10.1177/03000605251327478, PMID: 40302658 PMC12046155

[34] Bucuri CE Ciortea R Malutan AM Berceanu C Rada MP Mihu D. Progesterone’s serum level and a new ultrasonographic parameter in the first trimester pregnancy - prognostic factors for embryonic demise. Rev Bras ginecologia e obstetricia: Rev da Federacao Bras das Sociedades Ginecologia e Obstetricia. (2019) 41:525–30. doi: 10.1515/hmbci-2015-0030, PMID: 31546275

